# The two-component regulatory system CenK–CenR regulates expression of a previously uncharacterized protein required for salinity and oxidative stress tolerance in *Sinorhizobium meliloti*

**DOI:** 10.3389/fmicb.2022.1020932

**Published:** 2022-09-30

**Authors:** Eukene O. Bensig, Cecilio Valadez-Cano, ZiYu Kuang, Isabela R. Freire, Adrian Reyes-Prieto, Shawn R. MacLellan

**Affiliations:** ^1^Department of Biology and Environmental Science, University of the Philippines Cebu, Cebu City, Philippines; ^2^Department of Biology, University of New Brunswick, Fredericton, NB, Canada

**Keywords:** transcription, two-component regulatory, *cenK*, *chvI*, alpha-proteobacteria, salt stress, oxidative stress, thioredoxin

## Abstract

Genes of unknown function constitute a considerable fraction of most bacterial genomes. In a Tn5-based search for stress response genes in the nitrogen-fixing facultative endosymbiont *Sinorhizobium* (*Ensifer*) *meliloti*, we identified a previously uncharacterized gene required for growth on solid media with increased NaCl concentrations. The encoded protein carries a predicted thioredoxin fold and deletion of the gene also results in increased sensitivity to hydrogen peroxide and cumene hydroperoxide. We have designated the gene *srlA* (stress resistance locus A) based on these phenotypes. A deletion mutant yields phenotypic revertants on high salt medium and genome sequencing revealed that all revertants carry a mutation in genes homologous to either *cenK* or *cenR*. *srlA* promoter activity is abolished in these revertant host backgrounds and in a strain carrying a deletion in *cenK*. We also observed that the *srlA* promoter is autoregulated, displaying low activity in a wildtype (wt) host background and high activity in the *srl* deletion mutant background. The *srlA* promoter includes a conserved inverted repeat directly upstream of the predicted −35 subsequence. A mutational analysis demonstrated that the site is required for the high promoter activity in the *srlA* deletion background. Electromobility shift assays using purified wildtype CenR response regulator and a D55E phosphomimetic derivative suggest this protein acts as a likely Class II activator by binding promoter DNA. These results document the first identified CenK–CenR regulon member in *S. meliloti* and demonstrate this two-component regulatory system and gene *srlA* influences cellular growth and persistence under certain stress-inducing conditions.

## Introduction

Identifying genetic loci required for bacterial growth and persistence in varying physicochemical environments is an important pursuit. The nitrogen-fixing facultative endosymbiont *Sinorhizobium* (*Ensifer*) *meliloti* (Kuzmanović et al., 2022) has a large (6.7 Mb) tripartite genome (Galibert et al., 2001). This bacterium can persist as a free-living soil bacterium or as an intracellular legume symbiont where it undergoes physiological and morphological changes and begins fixing atmospheric nitrogen that can stimulate the growth of wild or agriculturally important host plants. These cellular lifestyles occur in very different environments and the soil environment particularly can involve widely varying physicochemical conditions. Accordingly, *S. meliloti* has been the subject of attempts to identify genes that when disrupted conditionally impair growth under stressful conditions that include high salinity (Wei et al., 2004; Domínguez-Ferreras et al., 2006; Miller-Williams et al., 2006; Vriezen et al., 2013; Calatrava-Morales et al., 2017), low pH (Reeve et al., 1998; Lagares et al., 2016; Albicoro et al., 2021), oxidative stress (Davies and Walker, 2007; Flechard et al., 2009; Lehman and Long, 2018) and other physical or chemical conditions.

Stress responses require linked sensory perception/transcriptional regulatory systems and in bacteria this requirement is largely met by three major protein classes: the Extracytoplasmic Function (ECF) σ factors and their associated sensory anti-σ factors (Lonetto et al., 1994; Helmann, 2002), one-component transcription factors (Ulrich et al., 2005), and two-component regulatory systems (TCS) that usually include an integral membrane sensory histidine kinase and cognate DNA-binding response regulator (Zschiedrich et al., 2016). The genome of *S. meliloti* includes genes encoding 11 ECF σ factors, over 40 TCSs and, relative to its genome size, an unusually large number (approximately 390) of one-component transcription factors (Ulrich et al., 2005). Given its facultative lifestyle, relatively large genome, agricultural importance, and its potential for linking sensory perception to transcriptional response, *S. meliloti* is a good candidate for investigating the regulatory mechanisms of stress responses.

A previous initiative to investigate the global transcriptional impact of deleting all 11 *S. meliloti* ECF σ factors (Lang et al., 2018) demonstrated that over 380 genes are collectively regulated by the ECF complement. In this work we describe the generation of a strain in which 10 of the 11 ECF σ factors have been deleted, including the general stress response σ factor, RpoE2 (Sauviac et al., 2007; Bastiat et al., 2010) that itself regulates the expression of at least 320 genes (Lang et al., 2018). This provides a genetic background in which an important sensory and transcriptional regulatory complement has been largely eliminated. Our long-term goal is to identify potentially redundant stress response genes in *S. meliloti*, where their physiological import is only manifest in a strain lacking the ECF σ factor complement.

In this initial attempt to pursue that strategy using our ECF σ factor-deficient strain as host, we employed Tn5 mutagenesis to identify novel genes conditionally important for survival on a high salinity stress-inducing media formulation. Here we show that the insertional inactivation or deletion of a gene of unknown function (locus *SMc03845*) renders cells unable to grow on solid medium supplemented with 0.16 M NaCl. The predicted protein encoded by this gene includes a thioredoxin-like fold and cells also display increased sensitivity to hydrogen peroxide and cumene hydroperoxide. Our subsequent analysis demonstrated that in this instance, the growth impairment phenotype was not related to the absence of the ECF σ factor complement, but this is nevertheless the first report of a phenotype associated with *SMc03845*, currently annotated as a conserved hypothetical gene of unknown function. We characterized the *SMc03845* promoter and show its activity is subject to both positive and negative regulation and is dependent upon CenK-CenR (Skerker et al., 2005; Lakey et al., 2022), a TCS that is emerging as an important regulator of cell envelope-related functions in the α-proteobacteria.

## Materials and methods

### Bacterial strains and plasmids

The bacterial strains and plasmids used in this study are listed in Supplementary Table S1. *S. meliloti* strains were cultured at 30°C and *E. coli* strains were grown at 37°C. Both species were grown on LB (Miller) broth or this medium solidified with 1.6% agar. For *S. meliloti*, LB broth was supplemented with 2.5 mM MgSO_4_ and 2.5 mM CaCl_2_. Other supplementations are as noted elsewhere.

### Unmarked deletion of ECF σ factor genes and other loci

Unmarked deletions in genes *rpoE*1–*rpoE*9 and ECF σ-like gene *SMc01150* (Supplementary Table S2) were generated using the *sacB* suicide vector pJQ200 (Quandt and Hynes, 1993). Briefly, 500–600 bp regions upstream and downstream of each gene were amplified and cloned into pJQ200 using the enzyme-free AQUA method (Beyer et al., 2015). Individually each plasmid was conjugated into strain RmP110 (Yuan et al., 2006) and single cross-over events were selected on LB medium supplemented with streptomycin (500 μg/ml) and gentamicin (60 μg/ml). Cells from a single colony were grown overnight without selection and plated on medium supplemented with 5% sucrose. From these plates several colonies were screened for sucrose resistance, gentamicin sensitivity, and these isolates were subjected to PCR with primers flanking the gene to identify those containing the required gene deletion. Deletion of all 10 ECF σ factor genes in strain NB1836 was confirmed using PCR and by whole genome sequencing. This strain does not include deletion of the ECF σ factor FecI, which a transcriptomic analysis (Lang et al., 2018) suggested regulates the expression of only 3 of the approximately 380 genes collectively regulated by ECF σ factors in *S. meliloti*.

Unmarked deletions of genes *srlA* and *cenK* were generated and confirmed by PCR as described above using primers listed in Supplementary Table S1, and by genome sequencing.

### Tn5 mutagenesis and screening for salt-sensitive mutants

To carry out mutagenesis of strain NB1836, 100 μl of an overnight culture was mixed with 100 μl of *E. coli* cells carrying pUT-miniTn5-Kn (Modal Inc.), spotted on an LB plate and incubated at 30°C overnight. After resuspension of the mating spot in 1 ml saline, 100 μl volumes were spread-plated on selective medium containing streptomycin (500 μg/ml) and neomycin (100 μg/ml). Typically, colonies arising from ten individual matings were resuspended in LB broth to generate a library of Tn5 insertion mutants. To screen for NaCl-sensitive insertion mutants, cells from the library stock were plated on 150 mm plates to a density of approximately 500 cfu/plate. After growth for 3 days, colonies were replica-plated onto plates containing growth medium supplemented with relevant antibiotics and this medium supplemented with 0.16 M NaCl. This concentration of NaCl was used based on preliminary trials in our laboratory using wt cells, where it delayed the rate of colony growth on solid medium (from 3 to 5 days) indicating stress, but ultimately did not prevent colony growth from single cells. Cells from colonies unable to grow on the salt-supplemented medium were selected for further analysis that included confirmation of the salt-sensitive phenotype and M12 phage transduction into the parental strain to confirm linkage of phenotype and insertion.

Arbitrary PCR was used to map the location of Tn5 insertions. Briefly, using purified genomic DNA as template, a combination of IS50 primers (Pr794 and Pr795) and degenerate primers (Pr791 and Pr796) were used in the first round of PCR. For the second round, nested primers (Pr793 and Pr797) were used. Amplified products extracted from agarose gels were subjected to Sanger sequencing using primers Pr793 and Pr797.

### Phenotype testing

*Sinorhizobium meliloti* strains were tested for salt-sensitivity on LB plates supplemented with 0.16 M NaCl. Typically, 5 μl from an overnight culture was spotted onto a plate containing the required antibiotics and streaked for isolated colonies. For testing sensitivity to oxidizing agents, 20 μl of an overnight culture was mixed with 5 ml of LB top agar (0.4% agar) and poured over a 100 mm petri plate containing 20 ml of solidified LB agar. Filter discs (40 mm) were supplemented with 10 μl of the stock oxidizing agent and placed onto the solidified top agar. Inhibition zones were measured after 24 h incubation at 30°C.

### Green fluorescent protein reporter gene assays

Transcription activity from gene promoters was estimated by measuring GFPUV fluorescence in a Spectramax M3 fluorimeter (Molecular Devices) with an excitation wavelength of 340 nm and emission wavelength of 510 nm. For the *srlA* promoter, we measured GFP fluorescence in cultures grown overnight (24 h) and diluted, and cultures grown to an O.D of 0.5 from an overnight culture with both strategies yielding similar results. For convenience, we therefore used diluted overnight cultures for all promoter activity assays. Typically, *S. meliloti* cells carrying derivatives of the *gfp* reporter vector pOT1 were grown for 24 h at 30°C with shaking at 200 rpm. Cells from a 1 ml volume were washed once in saline (0.85% NaCl) and diluted (1,3) in saline. 200 μl volumes were measured for fluorescence and optical density at 600 nm. Specific activity was calculated from arbitrary GFP fluorescence units divided by optical density.

### Determination of *srlA* transcription start site

The transcription start site of *srlA* was mapped using the protocol and components supplied by the 5´ RACE System for Rapid Amplification of cDNA Ends kit (Invitrogen). Briefly, *S. meliloti* strain NB1854 cells carrying plasmid pEB57 were grown to an OD_600_ of 0.5. Total RNA was purified using the protocol and components of the TRIzol Max Bacterial RNA Isolation kit (Ambion). Primers used for nested PCR amplifications are listed in Supplementary Table S1.

### Protein purification

Gene SMc03820 (*cenR*) DNA was amplified from RmP110 genomic DNA and ligated into pET17b generating pEB55. We used a Quikchange (Stratagene)-based site-directed mutagenesis protocol and primers Pr909 and Pr910 to change an aspartate codon at position 55 to a glutamate codon generating pEB56. Both the wt and D55E variant protein expression plasmids were transformed into *E. coli* BL21 (DE3; pLysS) cells. Expression of the proteins carrying a C-terminal hexahistidine tag was induced using 0.5 mM IPTG. A large fraction of protein formed inclusion bodies when expressed at 37°C. We therefore grew 1 l volumes at 37°C to an OD600 of 0.4 and transferred the volumes to 22°C before induction. After 18 h induction, cells were collected, subjected to 2 freeze–thaw cycles, resuspended in 20 ml lysis buffer (50 mM phosphate buffer [pH 7.5], 300 mM NaCl and 10 mM imidazole) and lysed using sonication. Insoluble material was pelleted by centrifugation for 20 min at 17,000 rpm and the clarified supernatant was supplemented with 0.5 ml (drained volume) of washed His-Bind Ni-NTA beads (Millipore). Protein binding occurred for 1 h at RT with slow rotation. After collection by low-speed centrifugation, beads were washed twice with 10 ml volumes of lysis buffer and transferred to a disposable chromatography column. To elute bound proteins, we used a step elution process (0.5 ml volumes, from 20 mM imidazole to 150 mM imidazole in 10 mM imidazole steps) and a final elution using 0.5 ml buffer containing 250 mM imidazole. Analysis by SDS-PAGE revealed essentially pure CenR protein in the 150 and 250 mM elution volumes. Buffer exchange to 50 mM phosphate (pH 7.5) lacking NaCl and imidazole was conducted using Amicon 10 kDa mw cutoff spin kits (Millipore). Protein concentration was determined using a Bradford-based protein assay (Bio-Rad) and bovine serum albumin (BSA) as standard.

### Electromobility shift assays

Double-stranded probe (60 bp, biotin labeled at each 5′ end) including the SMc03845 inverted repeat was purchased from IDT. A typical 14 μl binding reaction included: 50 mM phosphate buffer (pH 7.5), 0.5 μg BSA, 0.05 μg poly (dI-dC), 20 fmol labeled probe, and CenR concentrations as specified. When applicable, 2 pmol of unlabeled wt and mutant competitor DNA was used per binding reaction. Each binding reaction was incubated at RT for 20 m prior to separation on a pre-run 6% acrylamide minigel in 0.5 × TBE at 10°C. After transferring DNA to positively charged nylon membrane (BrightStar, Ambion) and UV crosslinking, the labeled probe was detected using the LightShift Chemiluminscent EMSA kit (Pierce). A Chemidoc MP Imaging System (Bio-Rad) was used for data collection.

### Genome sequencing and analysis

Genomic DNA from parental strains NB1854, NB1855 and six revertant derivatives of each parental strain was extracted using the GenCatch Genomic DNA Extraction Kit (Epoch Life Sciences). After measuring DNA concentration and quality (NanoVue Plus UV/Vis Spectrophotometer, GE), 150–300 ng/μl samples were submitted to The Center for Applied Genomics (TCAG; Toronto) for Nextera XT library preparation and whole genome sequencing (paired end, Illumina HiSeq2500). Quality checking of raw reads was performed using FastQC (Babraham Bioinformatics) analysis software followed by trimming using fastp (Chen et al., 2018). The trimmed reads were mapped to the *S. meliloti* Rm1021 reference genome (NCBI Accession number: ASM696v1) with Bowtie2 (Langmead and Salzberg, 2012) and the output (.sam file) was converted into .bam format using SAMtools.

As a comparator, an assembly for all sequence reads was performed *via* the SPAdes assembler tool using an in-house python script and the output files were aligned to the *S. meliloti* reference genome. MindTheGap and Ingap were used to detect single nucleotide polymorphisms (SNPs) or gene deletions. A visualization analysis was carried out using Geneious Prime 8.0.

### Other molecular biological techniques

Sanger sequencing services were provided by Genome Quebec (Montreal). Genome sequencing was conducted by The Centre for Applied Genomics at The Hospital for Sick Children (Toronto). Analysis was conducted using an in-house pipeline. *E. coli* transformation was conducted using cells rendered competent using the CaCl_2_ method. Transfer of plasmids *into S. meliloti* was accomplished using overnight triparental mating with *E. coli* strain MT616 carrying pRK600 as a helper plasmid. Site directed mutagenesis was accomplished using mutagenic oligonucleotides and a protocol based upon the Strategene QuikChange method (Agilent Technologies).

## Results

### Identification of a locus required for growth in high salt levels

**Figure 1:**
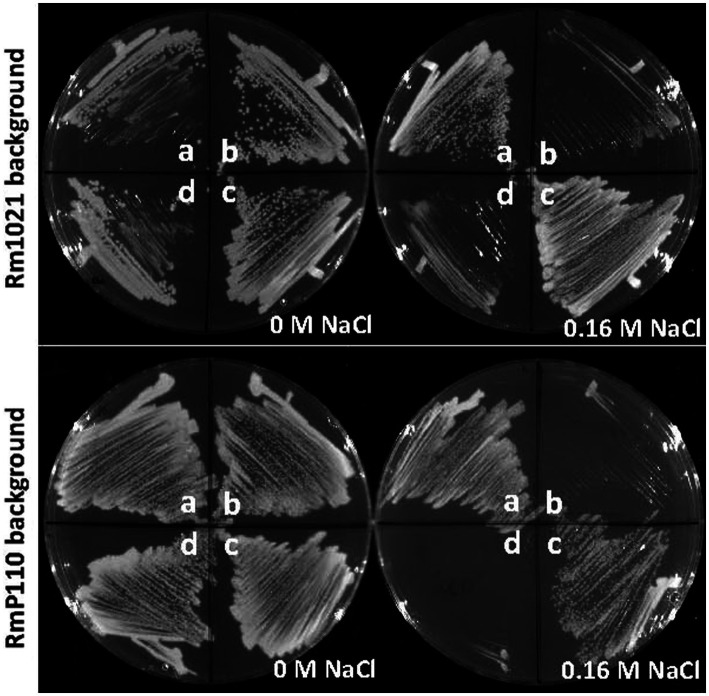
Salt-sensitive phenotype of *srlA* deletion strains. *srlA* was deleted in two wt host backgrounds: Rm1021 (top plates) and RmP110 (bottom plates) generating strains NB1903 and NB1854, respectively. Sectors a–d for all plates are as follows: **(A)**, wt strain; **(B)**, ∆*srlA* strain; **(C)**, ∆*srlA* strain complemented with pEB60; **(D)**, ∆*srlA* strain complemented with pEB66. Strains NB1903 and NB1854 fail to grow in LB medium supplemented with 0.16 M NaCl (sectors b). pEB60 carries the wt *srlA* ORF and complements the salt sensitive phenotype of a genomic *srlA* deletion (sectors c). pEB66 carries the *srlA* ORF with a 52 codon deletion and fails to complement the salt sensitive phenotype of a genomic *srlA* deletion (sectors d).

As part of a strategy to identify novel stress response genes we generated a strain of *S. meliloti* (NB1836; Supplementary Table S2) lacking most of its ECF sigma factors and used this as a host for mini-Tn5 transposon mutagenesis. After screening for growth defects on medium formulations that presented stressful growth conditions, we identified one insertion mutant that showed an inability to grow on LB (Miller) agar medium supplemented with 0.16 M NaCl. The transfer of neomycin resistance from the isolate to the parental strains NB1836 and RmP110 *via* transduction demonstrated that the salt-sensitive phenotype was linked to the Tn5 insertion. Using arbitrary PCR, we found that the transposon was inserted after nucleotide 89 in the open reading frame of gene *SMc03845*. Subsequently, the generation of an unmarked deletion of *SMc03845* in NB1836 (generating NB1855), confirmed that deletion of the gene resulted in the same salt-sensitivity observed in the original transposon insertion mutant. Subsequently, the generation of unmarked SMc03845 deletions in wt strains Rm1021 (generating NB1903) and RmP110 (Yuan et al., 2006; generating NB1854) showed the same salt-sensitive phenotype (Figure 1; plate sectors b). We amplified the *SMc03845* open reading frame (ORF) including 174 bp upstream of the ORF and ligated the product into plasmid pOT1 (Allaway et al., 2001). When this plasmid (pEB60) was conjugated into the two ∆*SMc03845* strains (NB1903 and NB1854), the salt-sensitive phenotype was complemented in each case (Figure 1; plate sectors c). The fortuitous presence of two unique PvuI sites in the *SMc03845* ORF enabled us to generate an in-frame 52 codon deletion in the gene carried by pEB60. When this plasmid (pEB66) was transferred into the ∆*SMc03845* strains, the salt-sensitive phenotype was no longer complemented (Figure 1; plate sectors d). Using the wildtype strains Rm1021 and RmP110 in this analysis demonstrated that the mutant phenotype was not related to the absence of ten of the ECF σ factors in strain NB1836 and most of the remainder of our characterization of this gene therefore involved only the parental strains RmP110 or Rm1021 and ∆*SMc03845* derivatives thereof. Based upon our phenotypic characterization of *SMc03845*, we have re-named *SMc03845 srlA* (*stress resistance locus* A).

### Phenotypic characterization of an *srlA* deletion mutant

**Figure 2:**
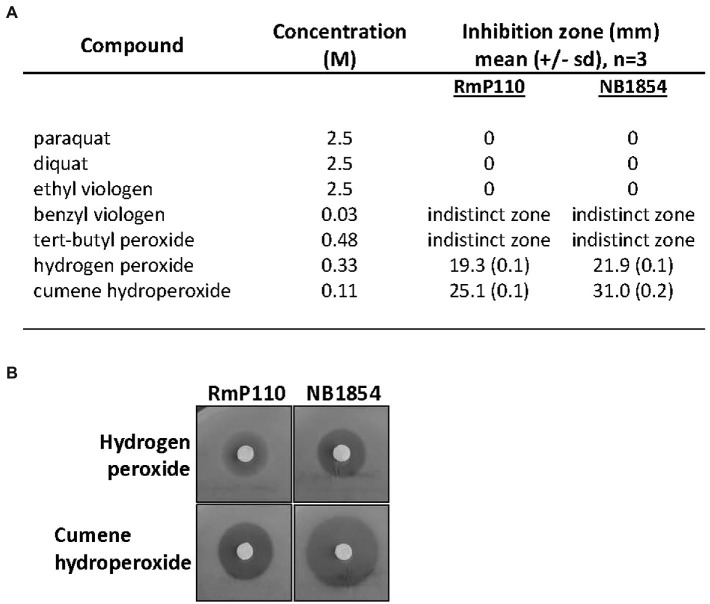
Effect of *srlA* deletion on resistance to oxidizing agents. **(A)** effects of compounds tested against wt strain RmP110 and ∆srlA strain NB1854. Benzyl peroxide and tert-butyl peroxide resulted in inhibition zones with indistinct margins that were difficult to measure. Mean differences between host strain zone diameters were significant for both hydrogen peroxide and cumene hydroperoxide (unpaired *t*-test, *p* < 0.0001). **(B)** representative disc experiments testing hydrogen peroxide and cumene hydroperoxide.

*srlA* is annotated as a conserved gene of unknown function and it is broadly distributed amongst the α-proteobacteria. A homolog of the protein from *Agrobacterium fabrum* strain C58 (protein Atu2684, 58% identity with SrlA) has been crystalized and structurally characterized (PDB 2AXO). We modeled the structure of SrlA from Atu2684 using the protein structure prediction program Phyre2 (Kelley et al., 2015) and Chimera (Pettersen et al., 2004). It carries a thioredoxin fold (Supplementary Figure S1) and is predicted to possess an N-terminal signal sequence. Although strain NB1854 (RmP110 [∆*srlA*]) is unable to grow on solid LB (Figure 1) supplemented with 0.16 M NaCl, we were unable to reproducibly detect a similar salt-sensitive growth defect during growth in liquid medium. Since SrlA appears to carry a redox-reactive thioredoxin fold, we tested sensitivity to a selection of inorganic and organic oxidizing agents. Relative to the parental strain RmP110, NB1854 demonstrated significantly (*p* < 0.0001) increased sensitivity to H_2_O_2_ and cumene hydroperoxide (Figure 2). Neither the parental strain nor the ∆*srlA* mutant showed sensitivity to paraquat, diquat, or ethyl viologen at the concentrations tested. Testing of benzyl viologen and *tert*-butyl hydroperoxide resulted in poorly demarcated inhibition zones that were difficult to accurately measure. No differential growth impairment was noted during growth at low pH (pH 5.6), elevated temperature (40°C), or on M9 minimal medium vs. LB medium for any of conditions tested (data not shown). Thus, the phenotypic consequences of *srlA* deletion appears rather restricted to elevated NaCl concentrations and certain oxidizing agents although our phenotypic characterization is continuing.

### Characterization of the *srlA* promoter

**Figure 3:**
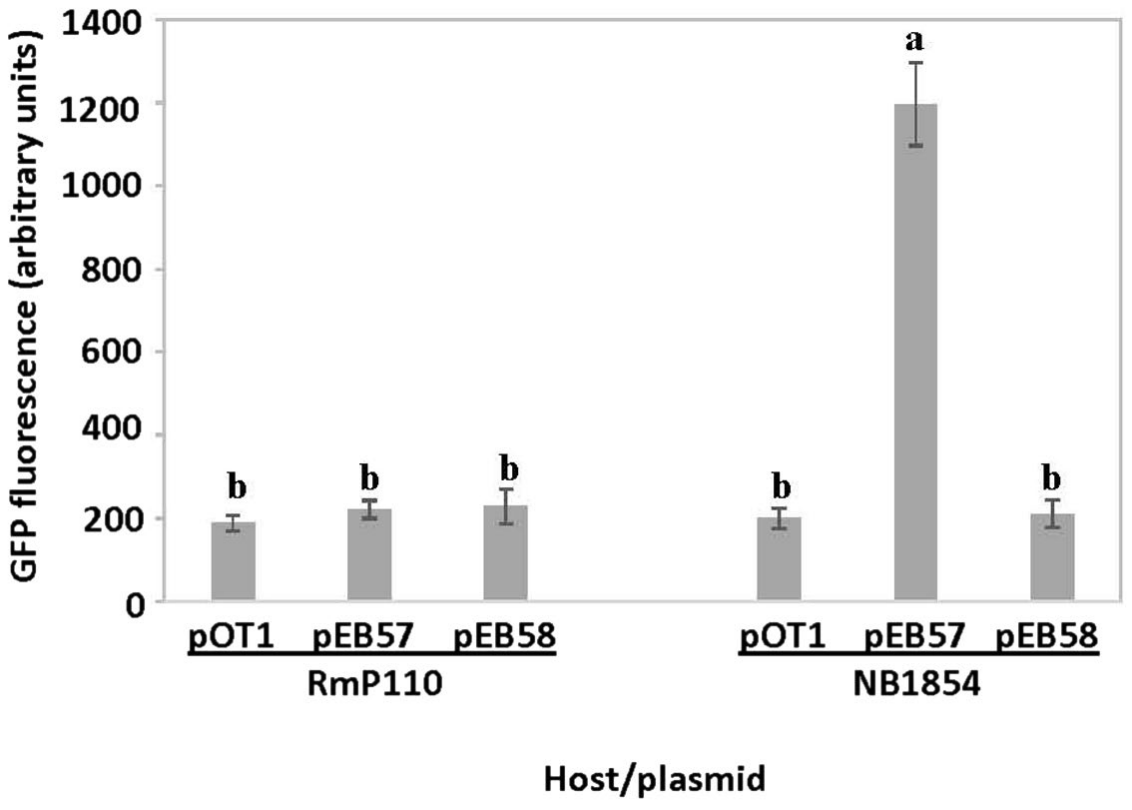
*srlA* promoter activity in wt and ∆*srlA* host backgrounds. Transcription activity detected from reporter vector pOT1, pEB57 (127 bp region upstream of *srlA* in same orientation as *gfp*) and pEB58 (127 bp region upstream of *srlA* in opposite orientation as *gfp*) in RmP110 (wt) and NB1854 (∆*srlA*) host strains. GFP activity reported as mean ± sd, *n* = 3. Letters indicate whether means are significantly different (*p* < 0.01, ANOVA and Tukey HSD).

**Figure 4:**
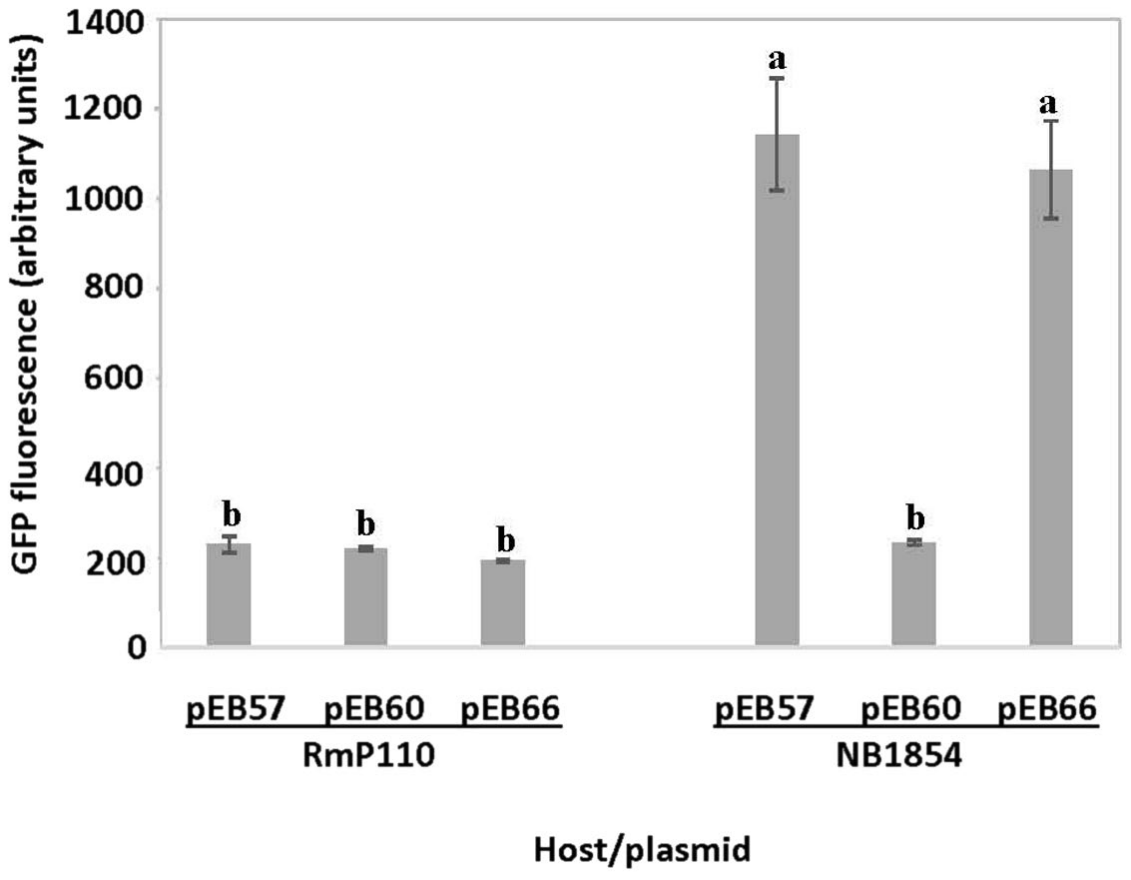
*in cis* repression of *srlA* promoter activity. Transcription activity detected from pEB57 (*srlA* promoter-*gfp* fusion) and pEB60 (*srlA* promoter-*srlA* ORF-*gfp* fusion) in RmP110 (wt) and NB1854 (∆*srlA*) host strains. pEB66 is a pEB60 derivative carrying an in-frame deletion in the *srlA* ORF. GFP activity reported as mean ± sd, *n* = 3. Letters indicate whether means are significantly different (*p* < 0.01, ANOVA and Tukey HSD).

We amplified a 127 bp region upstream of the *srlA* ORF and ligated the product into the GFP reporter vector pOT1 in both orientations generating plasmids pEB57 and pEB58. Only the construct with the *srlA* fragment in the same orientation as the *gfp* gene (pEB57) displayed significant (*p* < 0.01) promoter activity relative to the empty vector (Figure 3). This construct displayed low promoter activity in the parental RmP110 host but much higher activity in the ∆*srlA* host NB1854 suggesting an autorepression effect. To examine this more closely, we measured transcriptional activity from pEB60 (carrying the *srlA* promoter contiguous with its entire ORF). In the ∆*srlA* host this construct showed low promoter activity statistically indistinguishable from activity in the wt host (Figure 4). In contrast, the transcriptional activity from pEB66 (the pEB60 derivative with an in-frame 52 codon PvuI deletion in the *srlA* ORF) was high and statistically indistinguishable (*p* < 0.01) from promoter activity observed in the host cells carrying a genomic *srlA* deletion and just the promoter *in trans*. Thus, activity of the *srlA* promoter is low when cells harbor an intact *srlA* gene (either on the chromosome or on a plasmid), but high in the absence of *srlA* suggesting that protein SrlA negatively regulates (either directly or indirectly) its own transcription.

**Figure 5:**
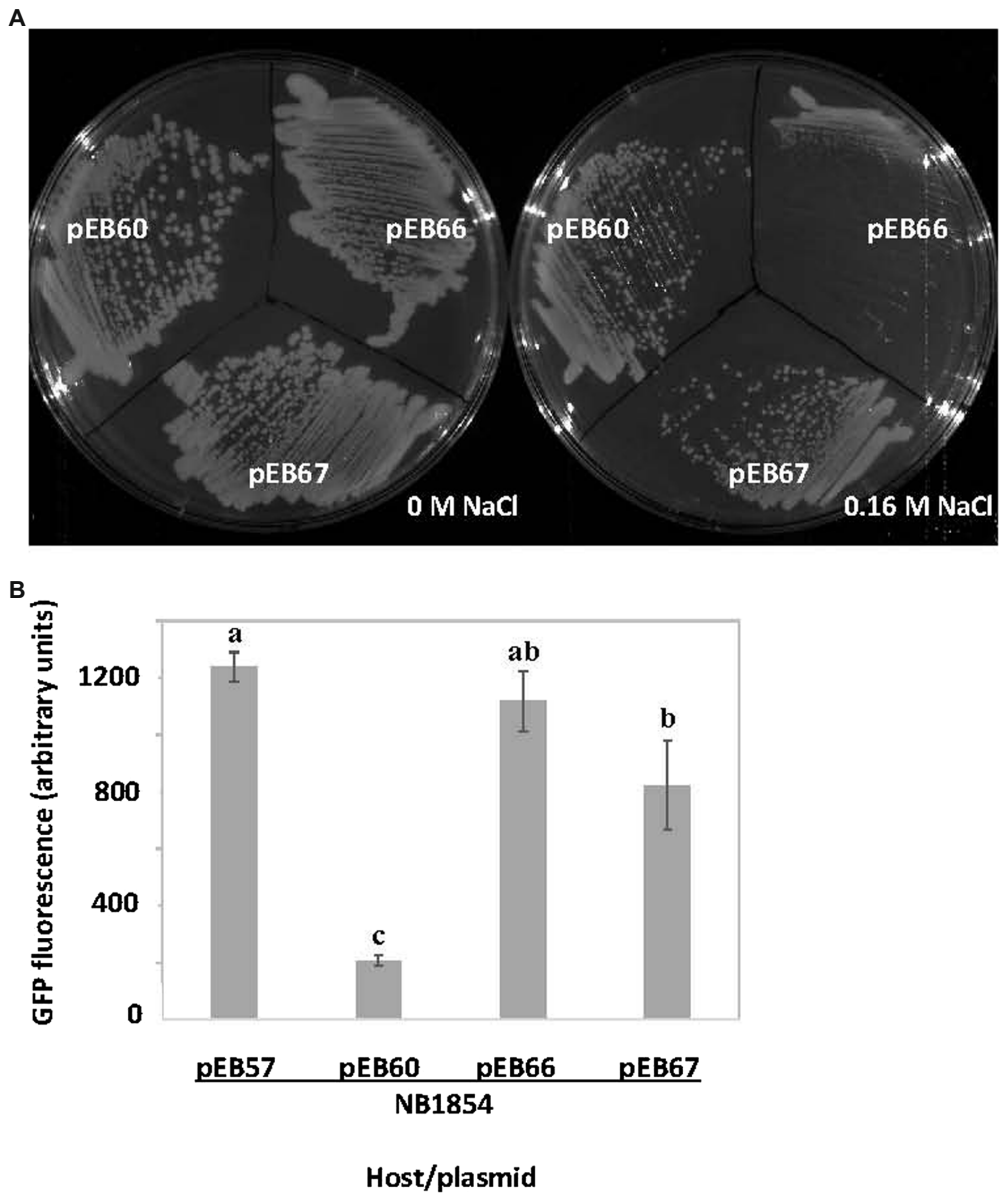
Effects of cysteine substitution mutation in SrlA thioredoxin fold. **(A)** the pEB60 (wt *srlA* ORF) derivative pEB67 carrying a C61S substitution mutation complements the salt-sensitive phenotype of ∆*srlA* strain NB1854. **(B)** the C61S SrlA substitution mutant expressed from pEB67 does not fully repress the *srlA* promoter as compared with expression of wt SrlA from pEB60. pEB66 is a pEB60 derivative carrying an in-frame deletion in the *srlA* ORF and can neither complement nor repress promoter. GFP activity reported as mean ± sd, *n* = 3. Letters indicate whether means are significantly different (*p* < 0.05, ANOVA and Tukey HSD).

SrlA carries a predicted thioredoxin-like fold (see Supplementary Figure S1). We found that a derivative carrying a C61S substitution mutation in its CXXC motif (pEB67) was still able to complement the salt-sensitive phenotype of a ∆*srlA* strain (Figure 5A) indicating that the derivative protein is expressed and functional. Interestingly however, the Cys substitution did diminish the autorepression phenotype (Figure 5B) yielding promoter activity that was statistically indistinguishable (*p* < 0.05) from the activity when repression was abolished by an in-frame deletion in plasmid-borne *srlA*.

**Figure 6:**
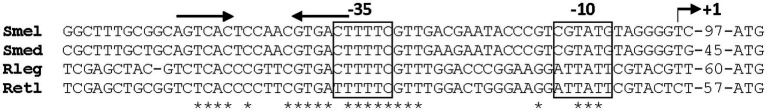
Alignment of *srlA* promoter region and analogous regions from other rhizobial genomes. *srlA* TSS (+1) from Schlüter et al. (2013) and experimentally verified (angled arrow) and distance to ATG start codon indicated. –10 and −35 subsequences (boxed) from Schlüter et al. (2013). Conserved inverted repeat indicated by arrows. Nucleotide conservation indicated by asterisks. Smel, *S. meliloti* 1,021 (locus SMc03845, *srlA*); Smed, *Sinorhizobium medicae* WSM419 (locus Sme_3082); Rleg, *Rhizobium leguminosarum* 3,841 (locus RL4535); Retl, *Rhizobium etli* CFN42 (locus RHE_CH03943).

**Figure 7:**
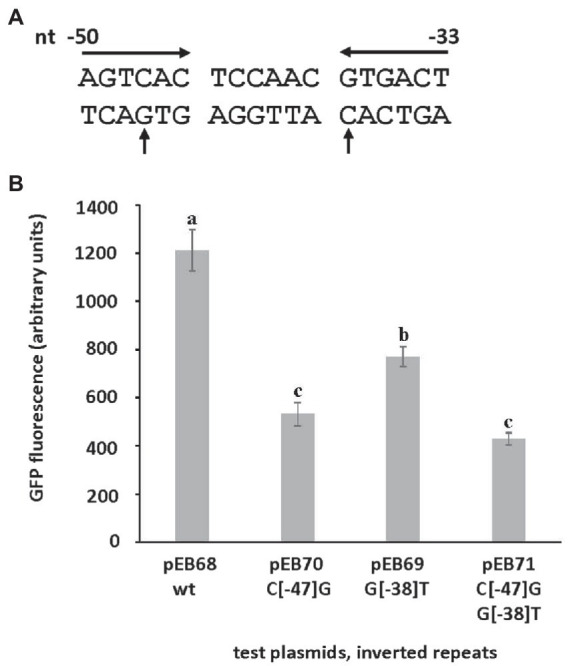
The effect of mutations in inverted repeat on *srlA* promoter activity. **(A)** *srlA* promoter repeat sequence (inverted arrows). Nucleotide numbering from + 1 TSS. Mutated nucleotides (C_−47_ and G_−38_) indicated (vertical arrows). **(B)** wt and mutant repeat *srlA* promoter activities in NB1854 host cells. GFP activity reported as mean ± sd, *n* = 3. Letters indicate whether means are significantly different (*p* < 0.01, ANOVA and Tukey HSD).

A likely transcription start site (TSS, + 1) for *srlA* was previously deduced in a global transcriptomic analysis (Schlüter et al., 2013). Since that study also indicated other possible (probably artifactual) TSSs for the locus, we used 5-RACE to experimentally determine the *srlA* TSS and confirmed its location 99 bp upstream of the start codon (Supplementary Figure S2). From previous data (Schlüter et al., 2013), this analysis, and a comparative sequence alignment using analogous regions from other rhizobial genomes, we were able to deduce the approximate location of the promoter −10 and − 35 subsequences (Figure 6). The comparative sequence alignment also revealed the presence of an inverted repeat (AGTCAC–NNCNNN–GTGACT) directly upstream and adjacent to the predicted *srlA*-35 subsequence (Figure 6). To assess the importance of the repeat to *srlA* promoter activity, we generated two mutations: C(−47)G in the left arm of repeat and G(−38)T in the right arm of repeat and compared activities with the wt repeat promoter. Both the C(−47)G mutation and, to a lesser extent, the G(−38)T mutation significantly (*p* < 0.01) reduced promoter activity relative to the wt promoter. The promoter carrying both mutations showed the least activity but was not statistically distinguishable from the construct carrying only the C(−47)T mutation. (Figure 7). These data, and the configuration and location of the repeat, suggest it is likely a recognition sequence for a Class II activator protein, a class of transcription factor that binds at or adjacent to the promoter −35 subsequence and activates transcription *via* protein–protein interaction with RNA polymerase holoenzyme (specifically, domain 4 of the σ factor subunit; Browning and Busby, 2016)

Using the perfect repeat found in the *srlA* promoter and allowing one mismatch, a computational search for similar inverted repeat sequences in the *S. meliloti* Rm1021 genome revealed three additional imperfect repeats in the predicted promoter regions of genes *SMc01226*, *SMc00517* and *chvI* (Table 1). In addition to strong conservation in the left and right arms of the repeats, all four share a conserved C nucleotide in the spacer region between the repeats. Interestingly, the inverted repeat in *SMc01226* is in the same position as the repeat in *srlA*—directly upstream and adjacent to the −35 subsequence. In contrast, in both *SMc00517* and *chvI*, the repeats are almost identically located close to their respective −10 subsequences and TSSs.

**Table 1:** Related inverted repeats in four promoter sequences.

Locus	Repeat sequence^a^	Distance from TSS (bp)^b^^,^^c^	Annotation^d^
*SMc03845* (*srlA*)	AGT**C**ACTCCAAC**G**TGACT	42	conserved hypo
*SMc01226*	ATTCACGACGTTGTGAAT	42	ArsR-family TF
*SMc00517*	AGTCACATCACTGTGACG	6	conserved hypo
*SMc02560* (*chvI*)	AGTCACGGCCGCGTTACT	0	response regulator
consensus	AgTCAC--C--cGTgAct		

a Perfect repeat (srlA) and imperfect repeats indicated in grey. srlA repeat nucleotides G(−38) and C(−47; bold) were mutated for promoter activity analysis (see Figure 7).

b Distance from approximate center of the inverted repeat to TSS.

c Transcription start site (TSS) assignment from Schlüter et al. (2013) and 5′-RACE for srlA (this work).

d Annotation from https://iant.toulouse.inra.fr/bacteria/annotation/cgi/rhime.cgi

### A reversion analysis reveals the regulatory role of a histidine kinase and response regulator

As described above, deletion of *srlA* results in the inability to form colonies on solid medium supplemented with 0.16 M NaCl, even after prolonged incubation. However, we frequently observed the emergence of a very few colonies on plates inoculated with high cell densities and these colonies formed with a growth rate similar to that of wt strain colony formation on the same high salt medium. We isolated several of these phenotypic revertants arising in two different ∆*srlA* host backgrounds: five from the NB1854 background and six from the ECF σ factor deletion strain (NB1855) in which we first detected the Tn5 insertional inactivation of *srlA*. Genome sequencing of these independently acquired revertants revealed the striking finding that every revertant possessed a mutation in either gene *SMc01716* (encoding a predicted histidine kinase) or in gene *SMc03820*, encoding a likely DNA-binding response regulator (Table 2). These proteins are homologs of the *Caulobacter crescentus* histidine kinase CenK and response regulator CenR and were previously predicted (Skerker et al., 2005) to comprise a likely two-component regulatory system in *S. meliloti*. The concordance of mutations in either *SM01716* or *SMc03820* in every revertant isolate (Table 2) suggests these proteins do indeed form a cognate pair of regulators in *S. meliloti*. Here we will refer to this *S. meliloti* TCS as CenK–CenR after the nomenclature used in *C. crescentus*. Discounting identical mutations within backgrounds (likely sibling effects) we documented four unique mutations (two in *cenK* and two in *cenR*) amongst the NB1854 reversion isolates and five unique mutations (three in *cenK* and two in *cenR*) in the NB1855 isolates (Table 2). Taken together, seven of the nine unique isolates possessed point mutations leading to amino acid substitutions in their respective proteins. Of the two without amino acid substitution mutations, revertant 2A had a single nucleotide substitution ten nucleotides upstream the *cenR* ATG start codon. This changes a likely ribosome-binding site sequence (GGAA) to GTAA and possibly reduces accumulation of CenR in the cell. Revertant 1B had a single nucleotide insertion causing a frameshift mutation early (after codon 85) in the 506 codon *cenK* gene (Table 2). The amino acid substitution mutations and their structural and functional consequences will be the subject of a future communication.

**Table 2:** Unique mutations detected in salt-resistant ∆*srlA* phenotypic revertant strains.

Background	Rev	Mutation^a^		Protein effect	Notes
		*SMc01716*	*SMc03820*		
NB1854					
	1A	T(1463)G	–	Ile488Arg	codon ATA → AGA
	2A	–	G(−10)T	RBS mutation?	10 nt before ATG
	3A	–	A(313)C	Lys105Gln	codon AAG → CAG
	5A	A(844)G	–	Ile282Val	codon ATC → GTC
NB1855					
	1B	A insertion	–	frameshift after codon 85	between nt 256/257
	3B	G(1375)A	–	Gly459Ser	codon GGC → AGC
	4B	–	A(476)G	Glu159Gly	codon GAA → GGA
	5B	–	T(284)C	Leu95Pro	codon CTC → CCC
	6B	G(1305)C	–	Met435Ile	codon ATG → ATC

a Unique mutations in the 1,521 bp (507 codon) *SMc01716* (cenK) gene and the 684 bp (228 codon) *SMc03820* (cenR) gene, number in bracket is nucleotide number of open reading frame (ORF).

**Figure 8:**
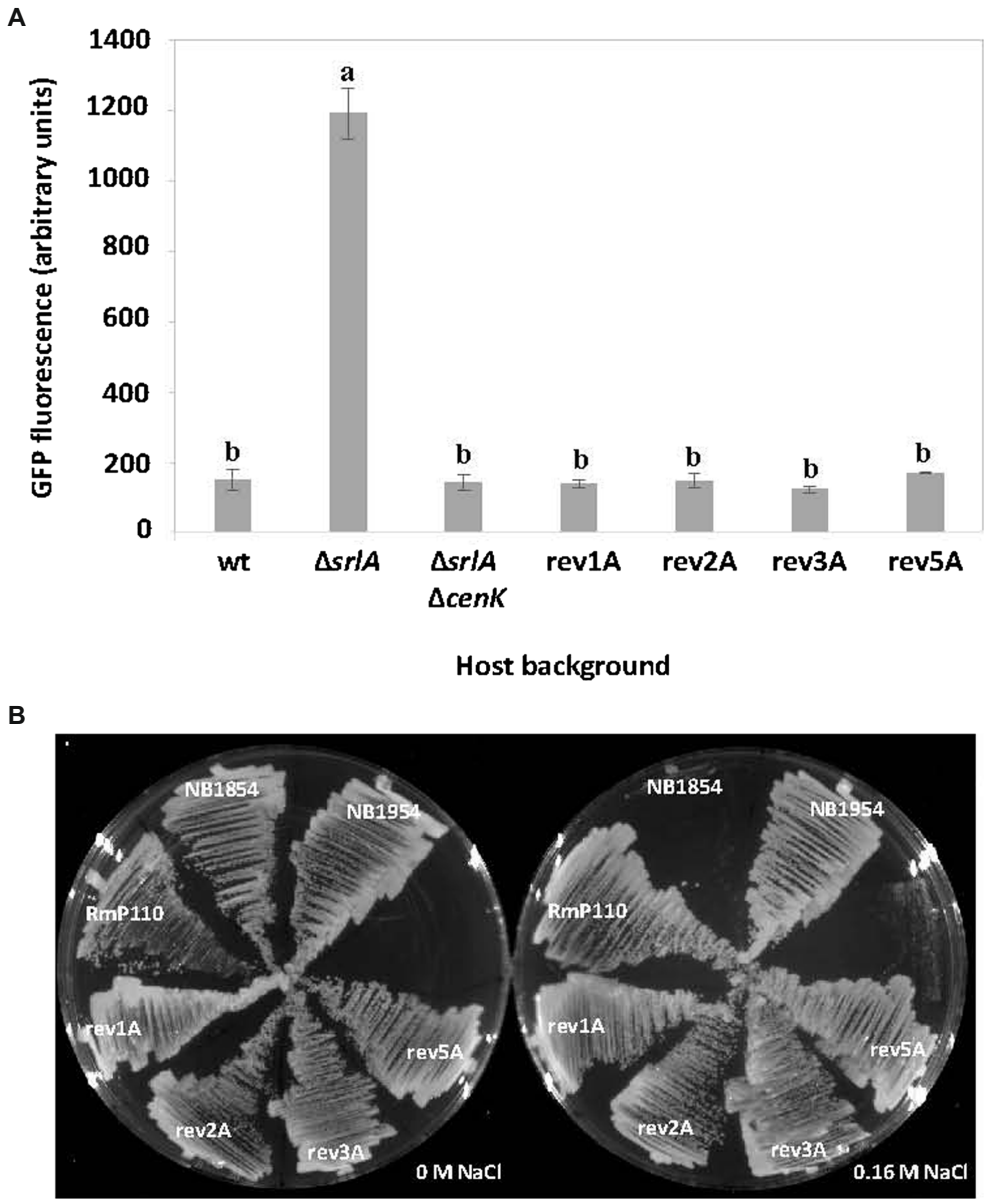
The effect of host *cenK* and *cenR* mutations on *srlA* promoter activity and salt-sensitivity. **(A)** transcription activity detected from *srlA*-*gfp* transcriptional fusion in plasmid pEB57 in genetic hosts: RmP110 (wt), NB1854 (∆*srlA*), NB1954 (∆*srlA* ∆*cenK*), and revertant strains 1A, 2A, 3A, and 5A (see Table 2 for revertant strain genotypes). GFP activity reported as mean ± sd, *n* = 3. Letters indicate whether means are significantly different (*p* < 0.01, ANOVA and Tukey HSD). **(B)** growth of strains on LB medium supplemented with 0 and 0.16 M NaCl.

Two lines of genetic evidence suggests that *S. meliloti* CenK-CenR regulates the expression of SrlA. First, while the *srlA* promoter-*gfp* transcriptional fusion in pEB57 displays high promoter activity in a ∆*srlA* background, the promoter activity is very low in a ∆*srlA* ∆*cenK* double mutant (Figure 8A) and is statistically indistinguishable (*p* < 0.01) from the low (repressed) promoter activity in the wt host background. Second, when we transferred the promoter-*gfp* reporter vector into the revertant strains 1A, 2A, 3A, and 5A (carrying the various point mutations in either *cenK* or *cenR*, Table 2) *srlA* promoter activity was also abolished in every instance (Figure 8A). These data demonstrate that CenK–CenR, either directly or indirectly, regulates transcription from the *srlA* promoter.

Finally, since mutations in either *cenK* or *cenR* cause reversion of the salt-sensitive phenotype of strain NB1854 we reasoned that an unmarked deletion of *cenK* in strain NB1854 should have the same effect. Indeed, the ∆*srlA* ∆*cenK* double mutant (NB1954) grows as well as the revertants and the wildtype strain on medium supplemented with 0.16 M NaCl (Figure 8B).

### CenR binds *srlA* promoter DNA

To ascertain whether the response regulator CenR directly regulates activity from the *srlA* promoter we expressed and purified (Supplementary Figure S3) hexahistidine-tagged wt CenR protein and a phosphomimetic mutant derivative of CenR, CenR^D55E^, and conducted electromobility shift assays (EMSAs) using *srlA* promoter DNA as a probe. The previously described inverted repeat centered at position −42 of the *srlA* promoter was centrally located in the 60 bp double-stranded DNA we used as probe (Supplementary Figure S4).

**Figure 9:**
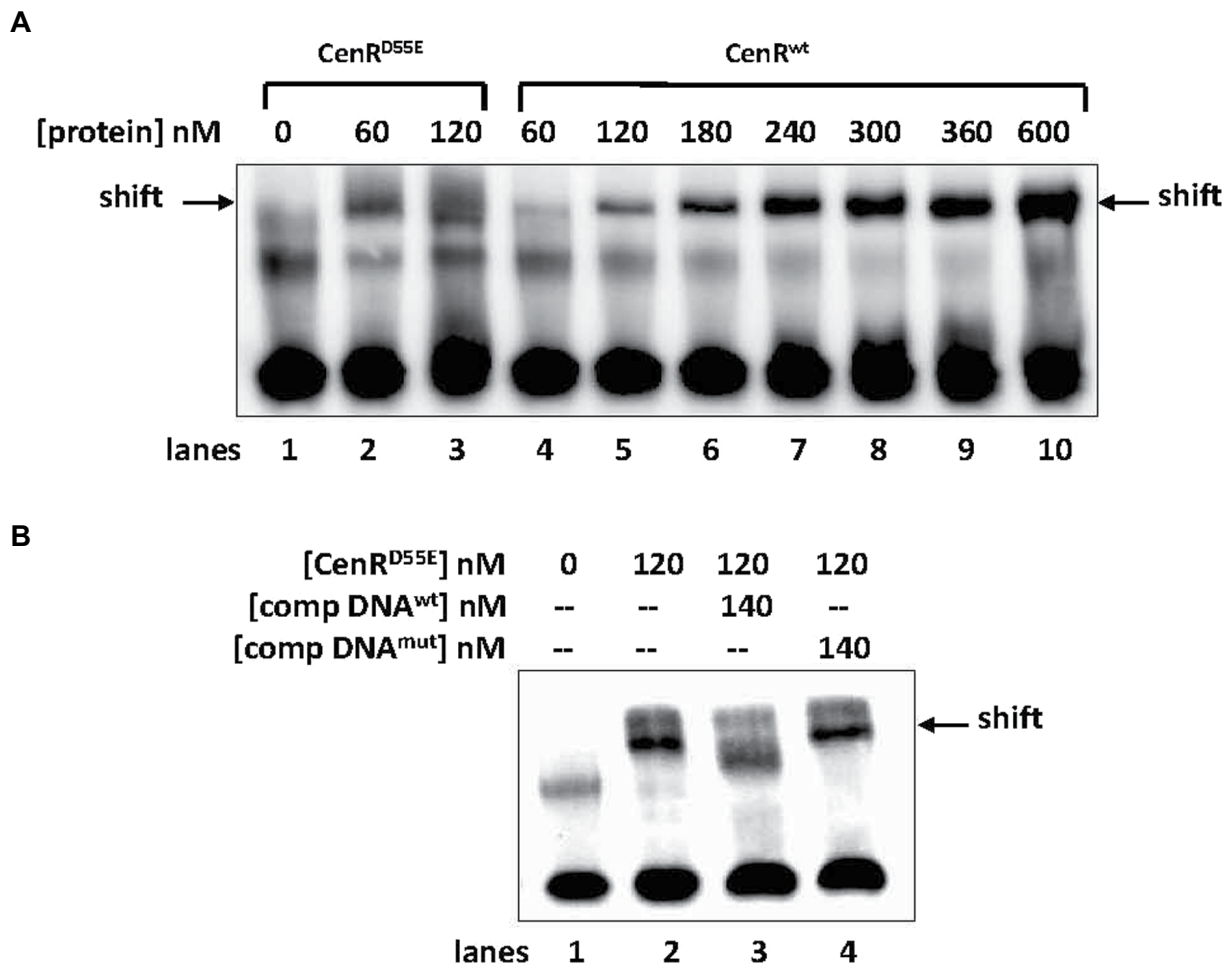
Binding of CenR protein to 60 bp *srlA* promoter region (see “Materials and methods”; Supplementary Figure S4). **(A)** Biotinylated probe (20 fmol) associated with no protein (lane 1) and increasing concentrations of purified (see Supplementary Figure S3) CenR^D55E^ (lanes 2–3) or CenR^wt^ (lanes 4–10) protein. **(B)** Probe binding challenged with unlabeled wt (lane 3) or unlabeled mutated DNA (lane 4).

Both CenR^wt^ and CenR^D55E^ bound and shifted probe DNA (Figure 9A) and at least qualitatively it appears as if CenR^D55E^ has a greater affinity for the probe than does CenR^wt^. The binding of CenR^D55E^ to *srlA* promoter DNA appears to be specific and dependent upon the inverted repeat sequence since challenging the binding with unlabeled DNA carrying the inverted repeat diminishes the observed shift (Figure 9B) but challenging with unlabeled DNA carrying a mutated inverted repeat does not.

## Discussion

Previous attempts to isolate salt-sensitive Tn5 mutants of *S. meliloti* identified a diverse group of genes that when disrupted led to an inability to tolerate elevated salt concentrations in growth media. In strain Rm1021, these genes included *SMb2012* (a putative DNA ligase), *fabG*, *pgm*, *exoF1*, *tig*, and *ftsE* (Miller-Williams et al., 2006) and in strain 042BM included genes *omp10* (a probable outer membrane lipoprotein), *greA*, *relA*, and *nuoL* (Wei et al., 2004).

Our attempt to detect salt-sensitive mutants resulted in the identification of an insertion in gene *SMc03845*, which led to increased sensitivity to elevated NaCl concentrations in growth medium, and sensitivity to certain oxidizing agents. This is the first attribution of a phenotype to gene *SMc03845* (*srlA*), a conserved gene of unknown function. To our knowledge, previous attempts to identify salt or oxidizing agent-sensitive mutants did not identify this locus. At least in the case of salt sensitivity, earlier attempts to identify mutants may have failed to detect *srlA* for two reasons. First, we used strain RmP110, a derivative of Rm1021 in which a frame-shift mutation in the phosphate transport gene *pstC* was repaired (Yuan et al., 2006). We note that while Rm1021 (∆*srlA*) is also salt-sensitive and unable to grow on medium supplemented with 0.16 M NaCl, its sensitivity is somewhat more muted than a RmP110 (∆*srlA*) derivative and most earlier screens for similar mutant phenotypes have used Rm1021 as host. Second, the salt-sensitive phenotype is only displayed during growth on solid medium, not in liquid culture, and this might also have contributed to not detecting *srlA* mutants in earlier screens. We are continuing our examination of SrlA to characterize its subcellular location, its role in the cell and the function of its thioredoxin-like domain, the phenotypic consequences of its deletion, and how its synthesis contributes to its transcriptional autorepression.

In addition to the autorepression phenotype we detected, our analysis of *srlA* promoter regulation also revealed that the CenK–CenR TCS is required for *srlA* promoter activity. The effect of deletion of a *cenR* homolog has been studied in *Brucella melitensis* (Zhang et al., 2009) and homologous CenK–CenR systems have been characterized in *C. crescentus* (Skerker et al., 2005) and *Rhodobacter sphaeroides* (Burger et al., 2017; Lakey et al., 2022) but not in *S. meliloti*. Lakey et al. (2022) predicted a recognition sequence for CenR binding in *R. sphaeroides* and other selected α-proteobacteria. This sequence (TGA–N8–TGA) aligns reasonably well with the inverted repeat sequence (AGTCAC–N6–GTGACT) we identified in *S. meliloti*.

Several of the results in this initial characterization seem enigmatic but hint at a more complex regulatory scheme that awaits description. First, it is surprising that impairment of a TCS system required for *srlA* transcription would also revert the salt-sensitive phenotype of a ∆*srlA* mutant. We are currently using genomic approaches to determine the CenK–CenR regulon but surmise that the loss of expression of one or more other regulon members in a CenK mutant leads to a compensatory phenotype that nulls the salt-sensitive phenotype of a *srlA* deletion mutant. Secondly, the response regulator CenR is essential in *C. crescentus* (Skerker et al., 2005) and *R. sphaeroides* (Burger et al., 2017; Lakey et al., 2022) and based on a Tn-Seq analysis (diCenzo et al., 2018) was predicted to be essential in *S. meliloti*. In accord with this prediction, despite repeated attempts we were unable to delete *cenR* although *cenK* could be readily deleted. This parallels the recent finding in *Rhodobacter sphaeroides* (Lakey et al., 2022) that *cenR*, but not *cenK*, is essential in that bacterium. Thus, although CenK and CenR are cognate, CenR must be able to retain its essential function in the cell in the absence of the kinase. Possibly, CenR can be phosphorylated by an alternative kinase or its essential function in the cell is mediated by the unphosphorylated form of the protein. Notwithstanding these possibilities, the deletion of CenK does abolish *srlA* promoter activity in *S. meliloti*, suggesting that phosphorylated CenR is required at this promoter in the cell. From *in vitro* EMSA experiments, it does qualitatively appear as if CenR^D55E^ binds srlA promoter DNA with greater affinity than CenR^wt^, but confirmation of this awaits a more quantitative analysis. Third, although CenR is essential in *S. meliloti*, we detected point mutations in *cenR* that leads to loss of *srlA* promoter activity and reversion of the salt-sensitive phenotype of a ∆*srlA* strain. Evidently, while these mutations in CenR debilitate its action at the *srlA* promoter, they are not lethally inactivating. Our current results suggest that CenR binds *srlA* promoter DNA with apparent specificity for the inverted repeat and this is consistent with our genetic examination of promoter activation. To strengthen the conviction that CenR directly acts upon the *srlA* promoter, we are currently continuing our DNA binding experiments and extending the analysis to the related repeats found in genes *SMc01226*, *SMc00517*, and *chvI* and will include purified CenR carrying the point mutations detected in our reversion analysis.

With respect to the similar promoter inverted repeats found in the *S. meliloti* genome, the differential location of these repeats, either adjacent to the −35 subsequences or near the TSS, suggests CenR can act either as an activator or repressor of transcription. It is worthwhile to note that while the cellular roles of proteins SrlA, SMc01226, and SMc00517 remain to be elucidated, ChvI is a particularly well-studied and important regulator of cell envelope-related functions in *S. meliloti* (Cheng and Walker, 1998; Bélanger et al., 2009; Chen et al., 2009; Bélanger and Charles, 2013; Ratib et al., 2018). The possibility this present work has identified an additional regulator of ChvI expression is intriguing.

Finally, the phenomenon of autorepression of the *srlA* promoter by SrlA expression also hints at a more complex regulatory network that awaits discovery. Although it is possible that SrlA directly binds *srlA* promoter DNA, it does not possess a recognizable DNA-binding motif and is predicted to possess an N-terminal signal sequence peptide. We are therefore exploring the possibility that secretion of SrlA stimulates a secondary signaling pathway that results in expression of a repressor protein that either acts directly upon the *srlA* promoter by occluding RNA polymerase binding, or by preventing the action of CenR at the *srlA* promoter. As documented in this work, a substitution mutation in one of the two cysteine residues in the thioredoxin fold of SrlA does not impair its ability to complement the salt-sensitive phenotype of a ∆*srlA* mutant but does diminish the autorepression effect. Whatever the primary role of SrlA in the cell and the importance of its thioredoxin-like domain to its function, this raises the possibility that at least the mechanistic path to autorepression involves a disulfide-dependent switch.

## Data availability statement

The original contributions presented in the study are included in the article/Supplementary material, further inquiries can be directed to the corresponding author.

## Author contributions

EB and SM conducted the experimental research and wrote the manuscript. ZK and IF contributed to experimental work. CV-C and AR-P contributed to genome sequence analysis and other computational analyses. All authors contributed to the article and approved the submitted version.

## Funding

This study was supported by a grant to SM from the Natural Sciences and Engineering Research Council of Canada (grant no. 386710). Molecular graphics were performed with the UCSF Chimera package. Chimera is developed by the Resource for Biocomputing, Visualization, and Informatics at the University of California, San Francisco (supported by NIGMS P41-GM103311).

## Conflict of interest

The authors declare that the research was conducted in the absence of any commercial or financial relationships that could be construed as a potential conflict of interest.

## Publisher’s note

All claims expressed in this article are solely those of the authors and do not necessarily represent those of their affiliated organizations, or those of the publisher, the editors and the reviewers. Any product that may be evaluated in this article, or claim that may be made by its manufacturer, is not guaranteed or endorsed by the publisher.
