# Supplementary material for: The two-component regulatory system CenK–CenR regulates expression of a previously uncharacterized protein required for salinity and oxidative stress tolerance in Sinorhizobium meliloti
Source: Front Microbiol. 2022 Sep 30;13:1020932. doi: 10.3389/fmicb.2022.1020932 (PMC9561847; doi:10.3389/fmicb.2022.1020932)
Supplement: Supplementary file 1 [file Table_3.DOCX]

Table S1. Strains, plasmids, and oligonucleotides used in this study.

| **Strains** |  | **Notes** | **Source** |
| --- | --- | --- | --- |
| ***E. coli*** |  |  |  |
|  | DH5α | *supE*44 ∆*lacU*169(φ80*lacZ*∆M15) *hsdR*17 *recA*1 *endA*1 *gyrA*96 *thi*-1 *relA*1 | lab strain |
|  | MT616 | helper strain, pRK600 | lab strain |
|  | NB187 | *E. coli* S17-1 λpir, pUT-miniTn5-Kn | Modal Inc |
|  | BL21 (DE3) pLysS | protein expression host | lab strain |
|  |  |  |  |
| ***S. meliloti*** |  |  |  |
|  | Rm1021 | spontaneous Sm^r^ isolate of strain SU47 | T. Finan |
|  | RmP110 | Rm1021 (*pstC* frameshift correction) | (Yuan et al., 2006) |
|  | NB1836 | RmP110, ∆*rpoE1*-*rpoE9*, *SMc01150* (*rpoE10*) | this work |
|  | NB1854 | RmP110 (∆*srlA*) | this work |
|  | NB1855 | NB1836 (∆*srlA*) | this work |
|  | NB1903 | Rm1021 (∆*srlA*) | this work |
|  | NB1954 | RmP110 ((∆*srlA* (∆*cenK*) | this work |
|  | Rev1A | NB1854 revertant (see Table 2) | this work |
|  | Rev2A | NB1854 revertant (see Table 2) | this work |
|  | Rev3A | NB1854 revertant (see Table 2) | this work |
|  | Rev5A | NB1854 revertant (see Table 2) | this work |
|  | Rev1B | NB1855 revertant (see Table 2) | this work |
|  | Rev3B | NB1855 revertant (see Table 2) | this work |
|  | Rev4B | NB1855 revertant (see Table 2) | this work |
|  | Rev5B | NB1855 revertant (see Table 2) | this work |
|  | Rev6B | NB1855 revertant (see Table 2) | this work |
| **Plasmids** |  |  |  |
|  | pJQ200 | *sacB* suicide vector | (Quandt and Hynes, 1993) |
|  | pUT-miniTn5-Kn | mini-Tn5 delivery vector | Modal Inc |
|  | pOT1 | *gfp* reporter vector | (Allaway et al., 2001) |
|  | pET17b | expression vector | Novagen |
|  | pEB55 | pET17b (Pr925-Pr926), CenR C-term His tag expression | this work |
|  | pEB56 | pEB55 sdm (Pr909-Pr910) CenR D55E | this work |
|  | pEB57 | *srlA* 197 bp promoter region (Pr927-Pr928) same orientation as *gfp* in pOT1 | this work |
|  | pEB58 | *srlA* 197 bp promoter region (Pr929-Pr930) opposite orientation as *gfp* in pOT1 | this work |
|  | pEB60 | *srlA* promoter + ORF (Pr927-Pr857) in pOT1 | this work |
|  | pEB64 | *cenR* Pr941-Pr942 (up)/Pr943-Pr944 (dwn) in pJQ200 | this work |
|  | pEB65 | *cenK* Pr945-Pr946 (up)/Pr947-Pr948 (dwn) in pJQ200 | this work |
|  | pEB66 | pEB60 with 52 codon PvuI deletion in *srlA* ORF | this work |
|  | pEB67 | pEB60 sdm (Pr903-Pr904) C61S SrlA | this work |
|  | pEB68 | *srlA* wt repeat (Pr963-Pr928) promoter-*gfp* fusion in pOT1 | this work |
|  | pEB69 | *srlA* mutated repeat (G[-38]T) (Pr964-Pr928) promoter-*gfp* fusion in pOT1 | this work |
|  | pEB70 | *srlA* mutated repeat (C[-47]G) (Pr965-Pr928) promoter-*gfp* fusion in pOT1 | this work |
|  | pEB71 | *srlA* mutated repeat (G[-38]T)/ (C[-47]G) (Pr966-Pr928) promoter-*gfp* fusion in pOT1 | this work |
| **Oligomers** |  |  |  |
|  | Pr243 | CTCCAGTGAAAAGTTCTTC |  |
|  | Pr857 | AAACTGCAGTCAGAGCTTTCCCTGTGGCC |  |
|  | Pr903 | CACCAGCCAGGGCAGCTCCTCCTGTC |  |
|  | Pr904 | GACAGGAGGAGCTGCCCTGGCTGGTG |  |
|  | Pr909 | cctcctgatcatggaggtaggtctgccggac |  |
|  | Pr910 | gtccggcagacctacctccatgatcaggagg |  |
|  | Pr927 | TATAAGCTTCGTGGGTTTTCCACGGCTTTG |  |
|  | Pr928 | ATACTGCAGGGCGTCTTTGTTCGTGCTCG |  |
|  | Pr929 | ATACTGCAGCGTGGGTTTTCCACGGCTTTG |  |
|  | Pr930 | TATAAGCTTGGCGTCTTTGTTCGTGCTCG |  |
|  | Pr941 | TCGATAAGCTTGATATCGAATTCCTGCAGCCCCCGTCGCGTCTATGCGGTCG |  |
|  | Pr942 | CGTGATCGATTCCTTTGTTTTG |  |
|  | Pr943 | CAAAACAAAGGAATCGATCACGGATTCCGTCCCTTCCCGGC |  |
|  | Pr944 | GTGGCGGCCGCTCTAGAACTAGTGGATCCCCCCGATTGGGACGGCGCCTTCCG |  |
|  | Pr945 | TCGATAAGCTTGATATCGAATTCCTGCAGCCCGTCGCGCATCATGCGTTCATG |  |
|  | Pr946 | GGGACAGTTCCTGTTGCTTCCTG |  |
|  | Pr947 | CACGGAAGCAAACAGGAACTGTCCCGCCGGACGATATGACCGGG |  |
|  | Pr948 | GTGGCGGCCGCTCTAGAACTAGTGGATCCCCCCTCGCAAGCCAGCTCGTCCG |  |
|  | Pr955 | CCGGATAACGGGAAAAG |  |
|  | Pr956 | CGGGCATGGCACTCTTG |  |
|  | Pr957 | GCGTTCCTGTACATAAC |  |
|  | Pr963 | TATAAGCTTCTTTGCGGCAGTCACTCCAACGTGACTTTTCGTTGACGAATACC |  |
|  | Pr964 | TATAAGCTTCTTTGCGGCAGTCACTCCAACTTGACTTTTCGTTGACGAATACC |  |
|  | Pr965 | TATAAGCTTCTTTGCGGCAGTGACTCCAACGTGACTTTTCGTTGACGAATACC |  |
|  | Pr966 | TATAAGCTTCTTTGCGGCAGTGACTCCAACTTGACTTTTCGTTGACGATATCC |  |
|  | Pr791 | GGCCACGCGTCGACTAGTCAGNNNNNNNNNNACGCC |  |
|  | Pr793 | GGCCACGCGTCGACTAGTCAG |  |
|  | Pr794 | AGGGCTTTACTAAGCTGATC |  |
|  | Pr795 | GGCGAAGTAATCGCAACATC |  |
|  | Pr796 | GCCACGCGTCGACTAGTCAGNNNNNNNNNNTCCGG |  |
|  | Pr797 | GGATGACCTTTTGAATGACC |  |
