# Supplementary material for: The two-component regulatory system CenK–CenR regulates expression of a previously uncharacterized protein required for salinity and oxidative stress tolerance in Sinorhizobium meliloti
Source: Front Microbiol. 2022 Sep 30;13:1020932. doi: 10.3389/fmicb.2022.1020932 (PMC9561847; doi:10.3389/fmicb.2022.1020932)
Supplement: Supplementary file 2 [file Table_4.DOCX]

Table S2. List of primers used to generate up and down flanking products for *sacB*-based deletion strategy of ECF σ factors (see Materials and Methods). Locus names indicate the distribution of genes across the three *S. meliloti* replicons. *S. meliloti* strain NB1836 carries unmarked deletion of all 10 σ factors. Deletions were confirmed by PCR and whole genome sequencing.

***rpoE1* (*SMc01419*)**

TCGATACGCTTGATATCGAATTCCTGCAGCCCCAACGGAATTTTGTGTTGTC

GCTCCCAACCGTGTTAACTCTTCCCCCATTCGCCCTTCACGCCG

GGCGAATGGGGGAAGAGTTAACACGGTTGGGAGCCCGACGAATTCGAAACGGTC

GTGGCGGCCGCTCTAGAACTAGTGGATCCCCCTCCCGTCTTCATACATCAGCATC

***rpoE2* (*SMc01506*)**

TCGATAAGCTTGATATCGAATTCCTGCAGCCCTCGATGTCGATCGCGATCAG

GTTCGGGACTTAACGCTTGCTAGTTGAAATGTTCCGAAGCGGAAC

GAACATTTCAACTAGCAAGCGTTAAGTCCCGAACTTCGATCCTGCAGCGCGGTC

GTGGCGGCCGCTCTAGAACTAGTGGATCCCCCAGGCATCAAGCACGAGGAAG

***rpoE3* (*SMc02713*)**

TCGATAAGCTTGATATCGAATTCCTGCAGCCCCGCGACAGGCACGCGCGACG

CAACCCTACTCATGCAATAAGTGCTGCTCTTCCAGTGAAACGTGC

TGAAGAGCAGCACTTATTGCATGAGTAGGGTTGCCGTTGCTGCAAACGAAGGG

GTGGCGGCCGCTCTAGAACTAGTGGATCCCCCGGCGGCGAGGAGCCTCGCGC

***rpoE4* (*SMc04051*)**

TCGATAAGCTTGATATCGAATTCCTGCAGCCCTTTTATGTCAAAGCCGCTGC

GGCCCTAGGGGTGGTAGACACTTGGACACCCGATCAGGGCTGCAC

GATCGGGTGTCCAAGTGTCTACCACCCCTAGGGCCACAGAAAACGACCTGAATCC

GTGGCGGCCGCTCTAGAACTAGTGGATCCCCCCATGAAGCAGGCCGTCGTCG

***rpoE5* (*SMb21484*)**

TCGATAAGCTTGATATCGAATTCCTGCAGCCCGGATAGGAGCCGCACGAAGC

CCCCTAAAACGGTACAGCTTCAGGGTCGAATATTGCCCTTCTGCC

CAATATTCGACCCTGAAGCTGTACCGTTTTAGGGGAGCGGACGAGCGGCTCTGTG

GTGGCGGCCGCTCTAGAACTAGTGGATCCCCCCATGCCTGCAAAGTCGAAGG

***rpoE6* (*SMa0143*)**

TCGATAAGCTTGATATCGAATTCCTGCAGCCCAGAGTCAGCCGGCGGGGAATG

CGTGAGCTCAACACTAACCCGGCCCTCCCGAAACTCCGCAATTTG

GTTTCGGGAGGGCCGGGTTAGTGTTGAGCTCACGACAAGAGCCGACTTCTCGG

GTGGCGGCCGCTCTAGAACTAGTGGATCCCCCGGCAGGCAACCGAAACCACTG

***rpoE7* (*SMb20531*)**

TCGATAAGCTTGATATCGAATTCCTGCAGCCCCCTCCACTTTCCGGCTCATC

GGCTAACCTCATAACTCGTGGATGCTGCACTTCCTCCCAATTCGC

GGAAGTGCAGCATCCACGAGTTATGAGGTTAGCCATATGGAAACGCAGGAACTCATC

GTGGCGGCCGCTCTAGAACTAGTGGATCCCCCATGGCCAAAATTGCCACCGC

***rpoE8* (*SMb20592*)**

TCGATAAGCTTGATATCGAATTCCTGCAGCCCGCATTCTGCTGACGGCCCCG

GTGGTAGACACTTGACACCTTGGGGGTCGGGCTCCTTCTCGGATATTG

GAGCCCGACCCCCAAGGTGTCAAGTGTCTACCACGCGGGAACGTTTCGAGCCGC

GTGGCGGCCGCTCTAGAACTAGTGGATCCCCCCTTCAATGCGGCCGCGACGT

***rpoE9* (*SMb20030*)**

TCGATAAGCTTGATATCGAATTCCTGCAGCCCTATCGATGCTCTCGCCAACC

CGAAAGTCCGCTACCCACCGGCGCCAGGCGACCGCCTTCTCGGCG

CGGTCGCCTGGCGCCGGTGGGTAGCGGACTTTCGAGCCTACCTTGAAAGCGCGCT

GTGGCGGCCGCTCTAGAACTAGTGGATCCCCCATGGCGAGGCCACATCAGCC

***rpoE10* (*SMc01150*)**

TCGATAAGCTTGATATCGAATTCCTGCAGCCCCCACACGACCGTGCTCCATG

AGTAAGTCGGCACCCGGGGTACTAGAGGGCAGCTCACCGGGTTTATAAAGC

AGCTGCCCTCTAGTACCCCGGGTGCCGACTTACTATTTTTTCTGGCCCCTGTCG

GTGGCGGCCGCTCTAGAAcTAGTGGATCCCCCCCGCATTCCTGCTCTCGGTA
