# Supplementary figures and images for: The two-component regulatory system CenK–CenR regulates expression of a previously uncharacterized protein required for salinity and oxidative stress tolerance in Sinorhizobium meliloti
Source: Front Microbiol. 2022 Sep 30;13:1020932. doi: 10.3389/fmicb.2022.1020932 (PMC9561847; doi:10.3389/fmicb.2022.1020932)

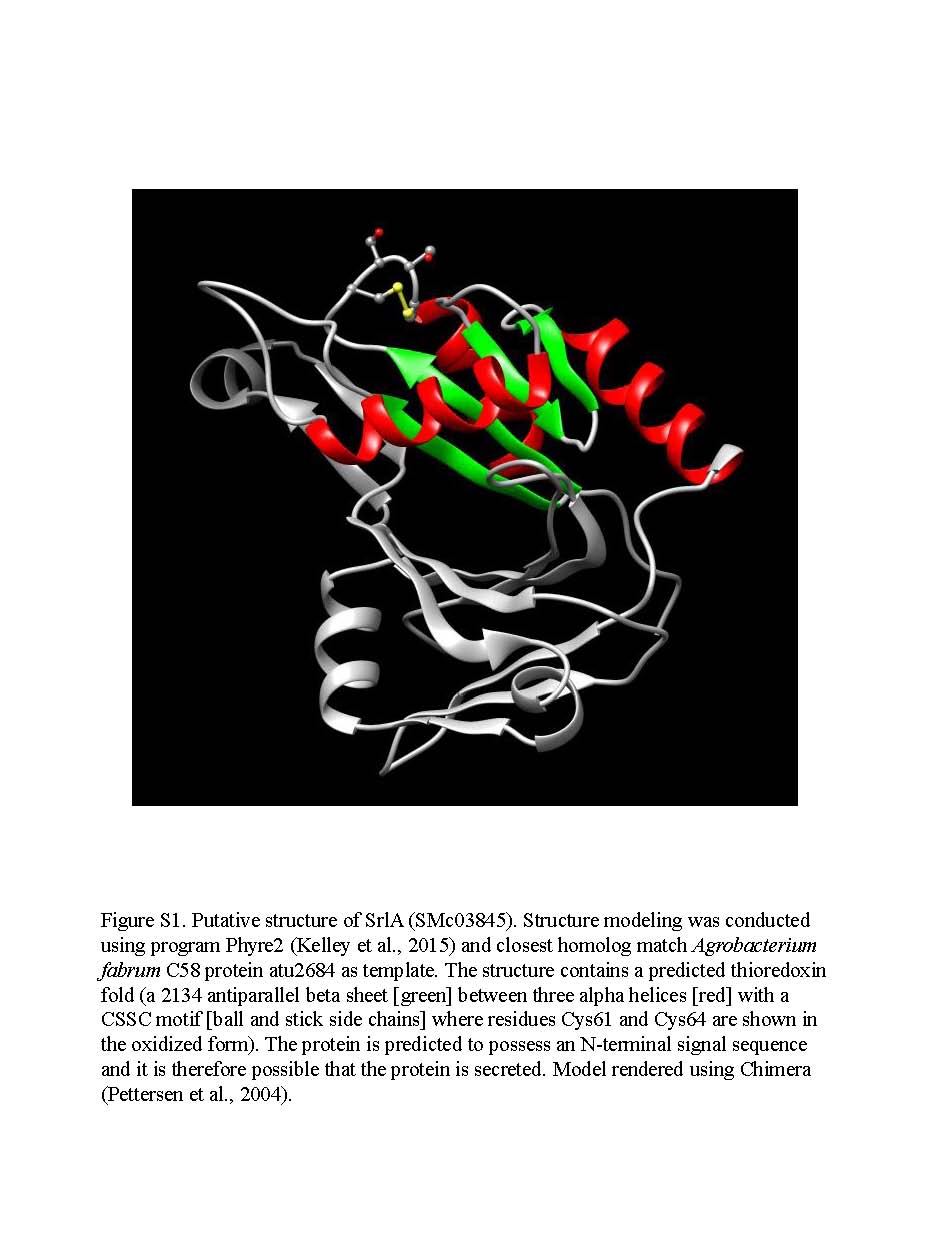

Supplement: Supplementary file 3 [file Image_1.JPEG]

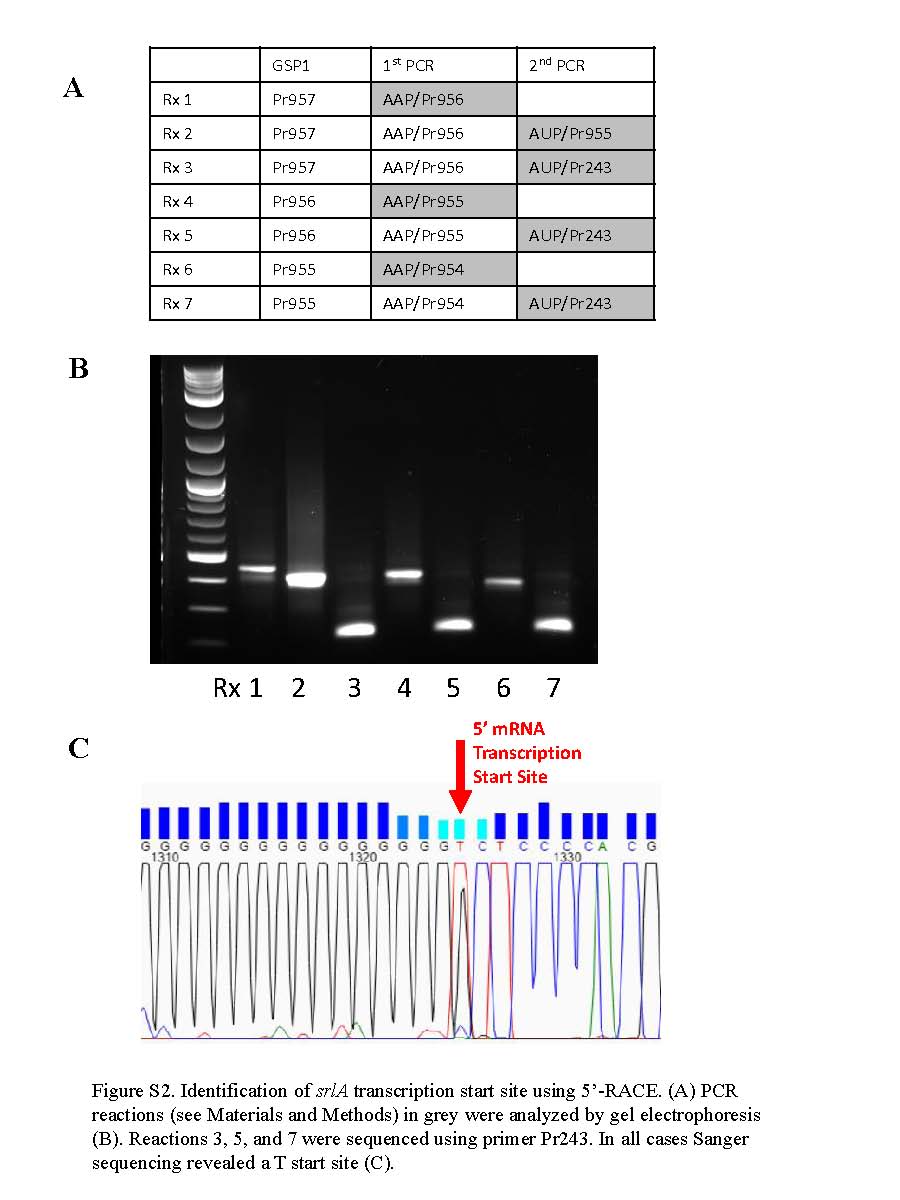

Supplement: Supplementary file 4 [file Image_2.JPEG]

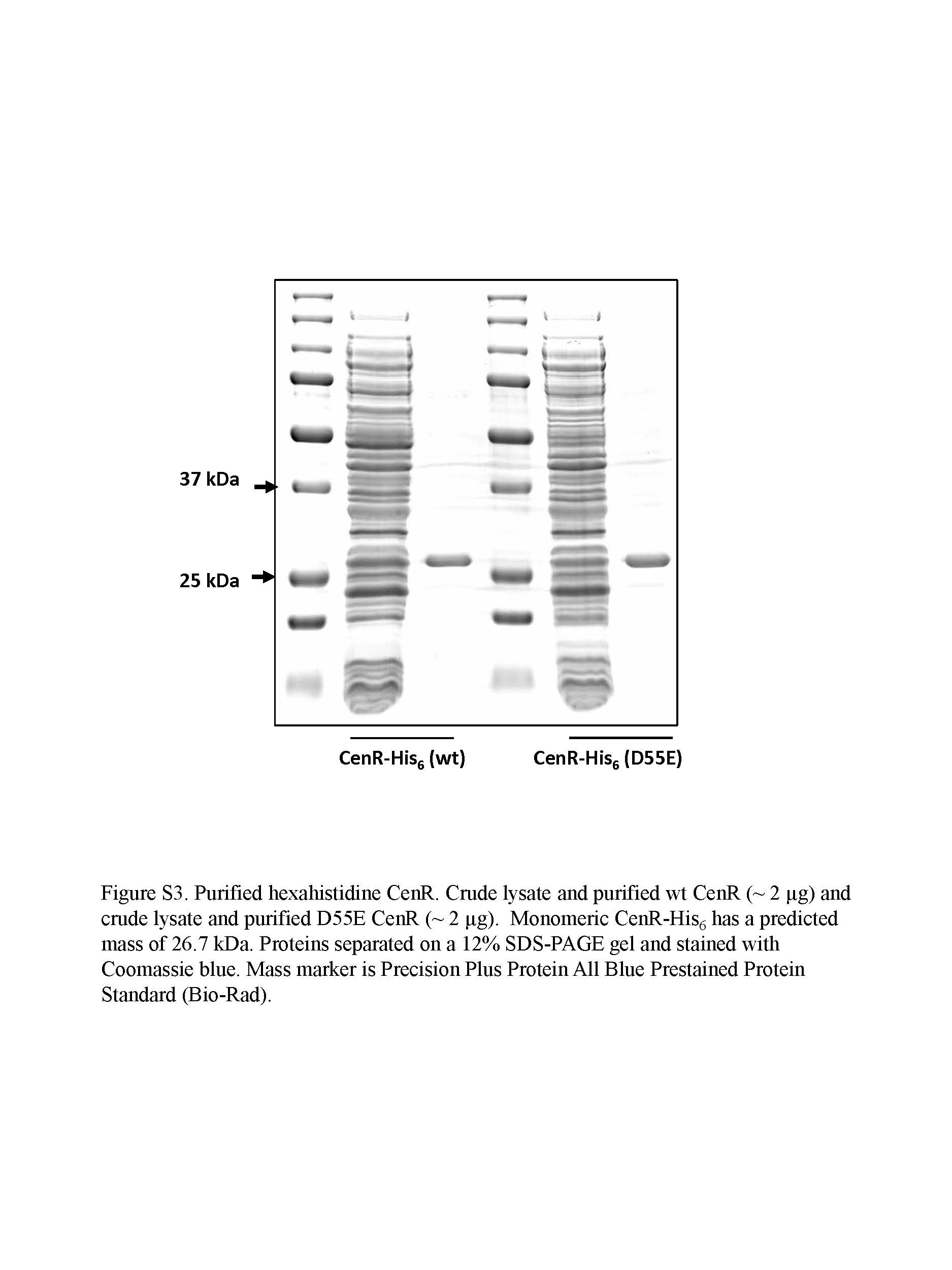

Supplement: Supplementary file 5 [file Image_3.JPEG]

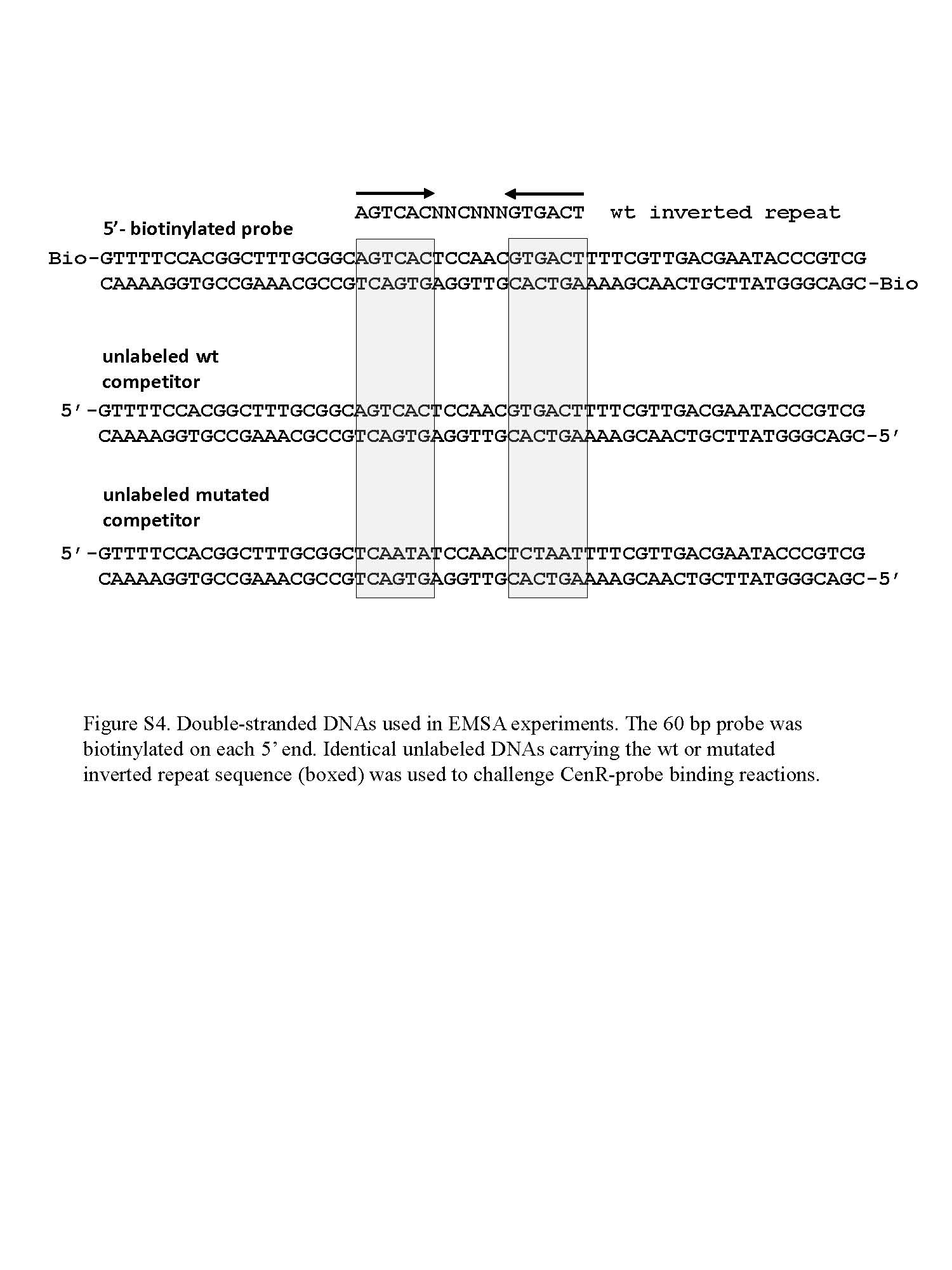

Supplement: Supplementary file 6 [file Image_4.JPEG]
